# Quinazolinone-Peptido-Nitrophenyl-Derivatives as Potential Inhibitors of SARS-CoV-2 Main Protease

**DOI:** 10.3390/v15020287

**Published:** 2023-01-19

**Authors:** Huynh-Nguyet-Huong Giang, Feng-Pai Chou, Ching-Yun Chen, Shen-Chieh Chou, Sheng-Cih Huang, Tuoh Wu, Bui-Thi-Buu Hue, Hong-Cheu Lin, Tung-Kung Wu

**Affiliations:** 1 Department of Material Science, National Yang Ming Chiao Tung University, 1001 Ta-Hsueh Rd., Hsinchu 30010, Taiwan; 2 Department of Biological Science and Technology, National Yang Ming Chiao Tung University, Hsinchu 30010, Taiwan; 3 Center for Emergent Functional Matter Science, National Yang Ming Chiao Tung University, 1001 Ta-Hsueh Rd., Hsinchu 30010, Taiwan; 4 Department of Chemistry, College of Natural Sciences, Can Tho University, Can Tho City 721337, Vietnam

**Keywords:** peptidomimetic, quinazolinone, main protease M^pro^, SARS-CoV-2, targeted covalent inhibitor

## Abstract

The severe acute respiratory syndrome coronavirus 2 main protease (SARS-CoV-2-M^pro^) plays an essential role in viral replication, transcription, maturation, and entry into host cells. Furthermore, its cleavage specificity for viruses, but not humans, makes it a promising drug target for the treatment of coronavirus disease 2019 (COVID-19). In this study, a fragment-based strategy including potential antiviral quinazolinone moiety and glutamine- or glutamate-derived peptidomimetic backbone and positioned nitro functional groups was used to synthesize putative M^pro^ inhibitors. Two compounds, **G1** and **G4**, exhibited anti-M^pro^ enzymatic activity in a dose-dependent manner, with the calculated IC_50_ values of 22.47 ± 8.93 μM and 24.04 ± 0.67 μM, respectively. The bio-layer interferometer measured real-time binding. The dissociation kinetics of **G1**/M^pro^ and **G4**/M^pro^ also showed similar equilibrium dissociation constants (*K*_D_) of 2.60 × 10^−5^ M and 2.55 × 10^−5^ M, respectively, but exhibited distinct association/dissociation curves. Molecular docking of the two compounds revealed a similar binding cavity to the well-known M^pro^ inhibitor **GC376**, supporting a structure−function relationship. These findings may open a new avenue for developing new scaffolds for M^pro^ inhibition and advance anti-coronavirus drug research.

## 1. Introduction

**Figure 1:**
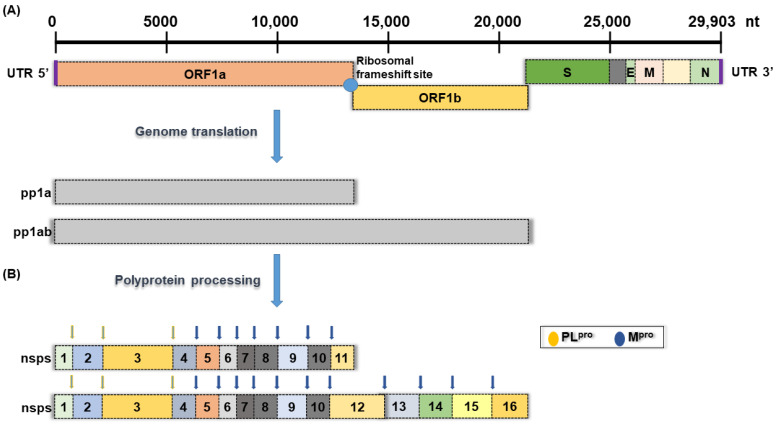
Schematic presentation of the SARS-CoV-2 genome organization. (**A**) Full-length genomic RNA (29,903 nt) that serves as an mRNA, containing ORF1a, ORF1b, and nine subgenomic RNAs. (**B**) Schematic representation of non-structural polyprotein cleavage sites. There are two viral proteases: a papain-like protease (PL^pro^) cleaves virus nonstructural polyprotein at three sites, and the other main protease (M^pro^) recognizes and cleaves the virus non-structural polyprotein at 11 sites.

The 2019−present global pandemic of coronavirus disease (COVID-19) has brought permanent changes to human daily life and interpersonal interactions around the world, as well as serious impacts and loss of economic activity. The disease is caused by a novel coronavirus called severe acute respiratory syndrome coronavirus 2 (SARS-CoV-2), which is more contagious than the closely related 2002 severe acute respiratory syndrome coronavirus (SARS-CoV) and 2012 Middle East respiratory syndrome coronavirus (MERS-CoV) infections [1]. Among various coronaviruses, SARS-CoV-2 and SARS-CoV enter host cells by binding to angiotensin-converting enzyme 2 (ACE 2), while MERS-CoV binds to dipeptidyl peptidase 4 (DPP4) receptors [2,3,4]. Coronaviruses are a group of enveloped single-stranded positive-sense RNA viruses with genomes ranging from 26 kb to 32 kb, encoding at least six open reading frames (ORFs) (Figure 1) [5]. Once inside the cell, genomic RNA is translated into nonstructural proteins (nsps) from two open reading frames (ORFs), namely ORF1a and ORF1b, and structural proteins from the rest of the RNA genome. ORF1a produces polyprotein 1a (pp1a) and is cleaved into 11 nsps. At the same time, a −1 ribosomal frameshift occurs upstream of the ORF1a stop codon and allows continued translation to ORF1b to generate a large polyprotein 1ab (pp1ab), which is cleaved into 15 nsps. Proteolytic cleavage is mediated by the viral proteases nsp3 and nsp5, which have a papain-like protease domain (PL^pro^) and a 3C-like protease domain (M^pro^ or 3CL^pro^), respectively (Figure 1) [5,6].

Current treatments for COVID-19 mainly focus on conserved proteins and enzymes, such as the spike protein (S), main proteases (M^pro^ or 3CL^pro^, nsp5), papain-like protease (PL^pro^), RNA-dependent RNA polymerase (RdRP, nsp12), and nucleoside-triphosphate hydrolase and helicase (NTPase/helicase, nsp13). For example, effective vaccines from Pfizer and Moderna, as well as antiviral drugs such as remdesivir and molnupiravir (MK-4482 and EIDD-2801), have been developed and have been widely available since the end of 2020 [7,8]. However, the failure of the public to comply with regulations and containment protocols, as well as the high mutation rate of the virus, has spawned multiple variants, namely Alpha, Beta, Gamma, Delta, and Omicron, which have higher transmission rates and/or antigen escape capabilities, resulting in breakthrough infections. Therefore, new treatments are still needed to reduce the risk of the progression of COVID-19 [7,8].

Main protease (M^pro^), also called 3C-like protease, is involved in its own replication, transcription, and maturation and has a role in viral entry into host cells [9,10]. It has unique substrate specificity and recognizes and cleaves 11 conserved glutamine residues (Leu-Gln↓(Ser, Ala, Gly)) in polyproteins. No known human protease has this substrate specificity, so the inhibition of M^pro^ activity blocks the replication of many coronaviruses with no adverse effect on human host cells. The essential functions of M^pro^ in the viral life cycle, conserved sequence and structural suitability, unique substrate recognition profile, and role in host cell entry make M^pro^ a promising drug target for the treatment of COVID-19. Up-to-date, many potential M^pro^ inhibitors have been screened based on structures, fragments, in silico, and databases [11] and references therein. On 21 December 2021, the FDA issued Emergency Use Authorization for Paxlovid, the first oral antiviral drug for the treatment of mild to moderate COVID-19 in adults and pediatric patients. Paxlovid consists of nirmatrelvir (PF-07321332), which targets the M^pro^ to stop viral replication, and ritonavir, which slows the former’s breakdown and helps it stay longer in the body at higher concentrations [12]. However, the use of Paxlovid with certain other drugs may cause a decrease in its own activity or an increase in blood levels of the other drugs, which may be harmful. In addition, the drug is also contraindicated in patients with severely reduced renal or hepatic function or in pregnant women. This suggests an urgent need to discover more M^pro^ inhibitors as drugs for COVID-19.

A compound design that integrates fragments with various functional structures has become a powerful tool for drug discovery. Quinazolinones are heterocyclic compounds bearing a carbonyl group on the C_4_N_2_ ring and are constituents of approximately 150 natural alkaloids that have shown attractive pharmacological activities, including antibacterial activity [13], antifungal activity [14], antimalarial activity [15], antiviral activity [13], anticancer activity [16], and sedative effects [17]. For example, febrifugine and its derivatives have been used to treat malaria, cancer, fibrosis, and inflammatory disease [18]. In parallel, the previous elucidation of putative M^pro^ inhibitors has revealed structural features such as peptidomimetic or amide structures and/or structural features containing aza, nitrile, amino, or nitro moieties as binding moieties to active site cysteine residues [19]. For example, the dipeptide-based M^pro^ inhibitor **GC376** is a broad-spectrum anti-coronavirus drug [20,21]. Furthermore, an example of the formation of a covalent nitrosothiol adduct by the active site Cys191 of *Mycobacterium tuberculosis* isocitrate lyase (ICL) with 3-nitropropionic acid shows that appropriately positioned weakly electrophilic groups forming covalent adducts with active site nucleophilic residues of proteins is an effective and rapid strategy for the development of designed inhibitors [22,23,24,25]. Combining a quinazolinone-derived antiviral activity, a non-human glutamine-specific recognition mode, and a nitro-directed covalent binding to the cysteine active site may yield highly active and specific M^pro^ inhibitors. In this study, we created a series of quinazolinone derivative compounds containing glutamine- or glutamate-derived peptidomimetic backbone and nitro/amino-functional derivatives to mimic M^pro^ substrate specificity and evaluated their effects on the inhibition of M^pro^ enzymatic activity. The results show that the synthesized compounds have obvious inhibitory activity against M^pro^ and their molecular docking results have better binding energies than **GC376** and are similar to the binding sites of the active region of the **GC376**−M^pro^ complex.

## 2. Materials and Methods

### 2.1. Synthesis of **G** Compounds

The synthesis of G compounds, **G1**–**G4**, is through intermediate **2a**, that is, isatoic anhydride (1 mmol, 0.16 g), L-glutamic acid (1.2 mmol, 0.177 g), potassium carbonate (4 mmol, 0.55 g), and 10 mL H_2_O were mixed in a round bottom flask at room temperature for 30 min, then iodine (1 mmol, 0.25 g) and formaldehyde (10 mmol, 0.37 mL) were added, and the mixtures were stirred at reflux for 6 h. After the reaction, the excess I_2_ was removed with 5 mL of saturated Na_2_S_2_O_3_, the reaction was partially purified, and the solution was neutralized to pH = 3 with a 10% HCl solution. The reaction mixture was extracted with 20 mL of ethyl acetate for 5 times, the organic layer was washed with a saturated NaCl solution, and the water was removed with anhydrous MgSO_4_. After the filtration and extraction of the organic layer, the crude yellow-orange product was obtained (234 mg, 85% yield).

To synthesize **G1**, **2a** (0.5 mmol, 138.12 mg), 4-dimethylaminopyridine (DMAP) (0.5 mmol, 61.09 mg), and 1-ethyl-3-(3-dimethylaminopropyl)carbodiimide (EDC) (1.5 mmol, 232.87 mg) were added to 10 mL of dichloromethane (DCM), cooled to 4 °C and stirred for 60 min before adding 3-nitroanilline (1 mmol, 138.14 mg), and the solution was stirred at room temperature for 4 h. The reaction solution was evaporated under vacuum, and the product was re-dissolved in 20 mL of ethyl acetate (EA) and then washed 3 times with 6 mL of ddH_2_O. The solvent in the organic layer was evaporated under vacuum, and the crude product was purified by column chromatography with an EA:Hex volume ratio of 2:1. The yield of **G1** was 70%.

^1^H NMR (400 MHz, DMSO-*d*_6_): δ 10.83 (s, 1H), 10.36 (s, 1H), 8.60 (t, *J* = 2.2 Hz, 1H), 8.51–8.45 (m, 2H), 8.13–8.08 (m, 1H), 7.99–7.92 (m, 2H), 7.86–7.79 (m, 2H), 7.74 (dd, *J* = 8.5, 2.0 Hz, 1H), 7.68 (d, *J* = 8.1 Hz, 1H), 7.62 (t, *J* = 8.2 Hz, 1H), 7.52 (td, *J* = 8.1, 2.1 Hz, 2H), 5.62 (dd, *J* = 10.0, 5.2 Hz, 1H), 2.66 (p, *J* = 6.9 Hz, 2H), 2.48–2.39 (m, 2H). ^13^C NMR (151 MHz, DMSO-*d*_6_) δ 170.80, 168.64, 160.78, 148.29 (d, *J* = 9.4 Hz), 147.86, 147.01, 140.50, 140.03, 135.03, 130.69, 130.43, 127.59, 126.73, 126.20, 125.32, 121.66, 118.81, 118.00, 114.44, 113.48, 57.60, 33.25, 26.04. The calculated electrospray ionization-high resolution mass (ESI-HRMS) of **G1** (C_25_H_20_N_6_O_7_) was 516.1393 g/mol. The experimental peak of *m*/*z* = 517.1472 was considered as [M + H]^+^.

To synthesize **G2**, **2a** (0.5 mmol, 138.12 mg), 4-dimethylaminopyridine (DMAP) (0.5 mmol, 61.09 mg), and 1-ethyl-3-(3-dimethylaminopropyl)carbodiimide (EDC) (1.5 mmol, 232.87 mg) were added to 10 mL of dichloromethane (DCM), cooled to 4 °C and stirred for 60 min before adding 4-nitrobenzyl alcohol (1 mmol, 153.40 mg), and the solution was stirred at room temperature for 4 h. The reaction solution was evaporated under vacuum, and the product was re-dissolved in 20 mL of ethyl acetate (EA) and then washed 3 times with 6 mL of ddH_2_O. The solvent in the organic layer was evaporated under vacuum, and the crude product was purified by column chromatography with an EA:Hex volume ratio of 2:1. The yield of **G2** was **75**%.

**G2**: ^1^H NMR (400 MHz, DMSO-*d*_6_) δ 8.42 (s, 1H), 8.25–8.04 (m, 5H), 7.87 (t, *J* = 7.4 Hz, 1H), 7.71 (d, *J* = 8.1 Hz, 1H), 7.57 (dd, *J* = 8.5, 2.4 Hz, 5H), 5.37 (s, 1H), 5.33 (s, 2H), 5.12 (s, 2H), 2.57 (dd, *J* = 16.1, 1.4 Hz, 4H). ^13^C NMR (151 MHz, DMSO-*d*_6_) δ 172.19, 169.09, 160.59, 147.97, 147.64, 147.58, 147.49, 144.17, 143.74, 135.31, 128.94, 128.91, 127.90, 127.74, 126.63, 123.92, 121.72, 65.96, 64.90, 58.91, 30.47, 24.56. The expected ESI-HRMS of **G2** (C_27_H_22_N_4_O_9_) was about 546.1387 g/mol. The experimental peak in (*m*/*z* = 547.1458) was considered as [M + H]^+^.

Synthesis of **G3**: In a 50 mL round bottom flask containing 5 mL of DMF, **2a** (0.5 mmol, 137.5 mg) and 4-nitro-3-aminophenol (1 mmol, 154.1 mg) were added followed by 100 μL of thionyl chloride via a syringe. The solution was stirred at room temperature for 5 min and then quenched with 10 mL of water. The solution was stirred for 30 min, and the precipitate was washed with 6 mL of water. The crude product was purified by silica gel column chromatography with an elution solution (EA:Hex volume ratio = 3:1). The yield of **G3** was 38%.

^1^H NMR (400 MHz, DMSO-*d*_6_) δ 10.40 (s, 1H), 10.32 (s, 1H), 10.21 (s, 1H), 9.90 (s, 1H), 8.36 (s, 1H), 8.18 (dd, *J* = 8.2, 1.5 Hz, 1H), 7.89–7.84 (m, 1H), 7.71 (dt, *J* = 8.2, 1.0 Hz, 1H), 7.58 (ddd, *J* = 8.1, 7.1, 1.2 Hz, 1H), 7.37 (d, *J* = 8.9 Hz, 1H), 7.29 (d, *J* = 2.8 Hz, 1H), 7.25–7.21 (m, 2H), 7.12–7.08 (m, 1H), 7.03 (dd, *J* = 8.8, 2.8 Hz, 1H), 5.56 (d, *J* = 9.5 Hz, 1H), 2.34 (q, *J* = 8.4, 5.8 Hz, 4H). ^13^C NMR (151 MHz, DMSO-*d*_6_) δ 170.21, 168.10, 160.68, 155.65, 155.11, 147.84, 146.71, 144.24, 144.05, 135.08, 128.64, 128.13, 127.65, 126.87, 122.76, 122.19, 121.76, 121.47, 121.31, 111.08, 56.81, 32.31, 29.46. The expected ESI-HRMS of **G3** (C_25_H_20_N_6_O_9_) was about 548.1292 g/mol. The experimental peak in (*m*/*z* = 549.1363) was considered as [M + H]^+^.

To obtain compound **G4**, **2a** (0.5 mmol, 137.5 mg) was added in a 50 mL two-necked round bottom flask containing 5 mL of DCM under argon. Then, thionyl chloride (100 µL) was added into the solution by a syringe, and the solution was kept at room temperature for 4 h. After removed the solvent, a dark brown oil product was obtained. The product was dissolved in 5 mL of a solution with a THF:TEA ratio of 1:1 and then cooled to 4 °C before adding 4-nitro-3-aminephenol (0.5 mmol, 77 mg). The solution was kept at room temperature for 30 min before quenched with 6 mL of ddH_2_O. The product was extracted with EA (10 mL) and washed with ddH_2_O (6 mL). Following the removal of the solvent, the crude product was purified with column chromatography with an elution solution (EA:Hex volume ratio = 2:1). The yield of **G4** was 42%.

^1^H NMR (400 MHz, Methylene Chloride-*d*_2_) δ 8.32 (d, *J* = 8.0 Hz, 1H), 8.15 (s, 1H), 7.86–7.78 (m, 3H), 7.76 (d, *J* = 8.0 Hz, 1H), 7.56 (s, 1H), 7.19 (dd, *J* = 9.1, 2.8 Hz, 1H), 7.13 (dd, *J* = 9.1, 2.7 Hz, 1H), 6.85 (dd, *J* = 9.1, 6.0 Hz, 2H), 6.11 (s, 4H), 5.39 (dd, *J* = 9.2, 5.5 Hz, 1H), 2.91–2.84 (m, 1H), 2.79–2.68 (m, 2H), 2.62–2.55 (m, 1H). The expected mass of **G4** (C_25_H_20_N_6_O_9_) was about 548.1292 g/mol. ^13^C NMR (151 MHz, DMSO-*d*_6_) δ 171.68, 168.52, 160.71, 148.06, 147.72, 145.08, 144.82, 139.23, 139.04, 135.39, 131.10, 130.76, 129.36, 128.02, 127.82, 126.67, 121.75, 120.62, 120.27, 58.98, 30.46, 24.51. The ESI-HRMS experimental peak in (*m*/*z* = 549.1366) was considered as [M + H]^+^.

### 2.2. Molecular Cloning and Protein Expression of M^pro^

The SARS-CoV-2 M^pro^ gene from Addgene was constructed on the vector pGEX-5X, which was linked to a glutathione S-transferase (GST) tag at the *N*-terminus of the recombinant M^pro^ protein. The recombinant plasmid pGEX-5X−SARS-CoV-2−M^pro^ was transformed into *E. coli* BL21 (DE3) for protein expression. Cell cultures were spread on LB agar plates containing 100 μg/mL ampicillin and incubated overnight at 37 °C. The single colony was picked and transferred to a 3 mL LB medium tube supplemented with 100 μg/mL ampicillin and incubated at 37 °C at 220 rpm for 16 h. The overnight culture was diluted into 1 L LB broth (volume ratio: 1:100) and incubated at 37 °C and at 220 rpm until OD_600_ reached 0.6–0.7. Isopropyl thio-β-Dthiogalactopyranoside (IPTG) (0.5 mM) was added to induce M^pro^ protein expression for 10 h at 16 °C and 180 rpm. Cells were pelleted at 6500 × *g* for 30 min at 4 °C and resuspended in 20 mL lysis buffer for protein purification.

The suspension was sonicated for 1 h at a 30% energy using a Vibra-Cell™ Ultrasonic Liquid Processors VCX 500, pulsing 1 s “on” and 3 s “off” on the ice. Cell lysate was centrifuged at 10,000× *g* for 40 min at 4 °C to separate the supernatant and the pellet. The supernatant was filtered through a 0.45 µm pore size filter to obtain a crude solution. To purify M^pro^, the crude solution was mixed with 1 mL of Glutathione Sepharose 4 Fast Flow resin and incubated for 12 h at 4 °C by shaking. Afterwards, the resin was washed 10 times with 10 mL of lysis buffer and re-suspended in 500 µL of lysis buffer and 10 µL of Factor Xa. The mixture was incubated at 4 °C for 36 h. The M^pro^ was collected by flow-through fractions and analyzed by SDS-PAGE.

### 2.3. In Vitro M^pro^ Inhibition Assay

M^pro^ activity assays were performed in a 384-well black flat-bottomed microtiter plate (Thermo Scientific™ Nunc™) in a final volume of 25 μL. Non-GST-tagged M^pro^ protein was added at a final concentration of 50 nM at 37 °C with various concentrations (3.125, 6.25, 12.5, 25, 50, and 100 μM) of compounds and assay buffer (final concentration: 20 mM Tris, 100 mM NaCl, 1% DTT, 1% EDTA, and 1% DMSO) for 30 min. The FRET substrate DABCYL-KTSAVLQSGFRKME-EDANS (50 µM) was added, and the solution was incubated at 37 °C for 1 h. Blank wells had the same compound concentrations as the substrate but did not contain M^pro^ protein. Inhibition was calculated by comparison to control wells to which no inhibitor was added. Fluorescence signals (excitation/emission: 355 nm/460 nm) of the released EDANS were measured using a fluorometer (Fluoroskan Ascent FL). IC_50_ values were determined by nonlinear regression (GraphPad Prism 8.0.1). For calculations, 100% active enzyme was assumed.

### 2.4. Bio-Layer Interferometry Binding Kinetics Assay

Label-free bio-layer interferometry (BLI) assays were performed by the Octet K2 two-channel system (FortéBio) at the Center for Emergent Functional Matter Science, National Yang Ming Chiao Tung University. BLI measurements were performed at a shaking speed of 1000 rpm and a temperature of 30 °C. A phosphate buffer with 0.1% Tween-20 (PBS-t) was a kinetic buffer. To prepare M^pro^ binding test probes, Ni-NTA optical fiber probes were run at baseline for 60 s, loaded into 200 µL of 50 µg/mL M^pro^-His diluted in PBS-T for 600 s and then run again at baseline in PBS-T for 60 s. The M^pro^-loaded probes were stored in PBS-T at 4 °C until kinetic analysis. For kinetic binding assays, serial dilutions of five concentrations of **G** compounds in PBS-T were added to one row of wells in a black polypropylene 96-well microplate (Greiner Bio-one), and one row of wells filled with PBS-T was used as a reference control in parallel. One assay cycle consisting of 60 s of baseline normalization in PBS-T and 150 s of association in compound solution, followed by 150 s of dissociation in PBS-T and 30 s of regeneration in 10 mM glycine buffer (pH 1.7) was performed for each compound concentration with M^pro^-loaded probes and blank probes. BLI results were analyzed using FortéBio Data Analysis High Throughput 12.0. The curves were aligned with the dissociation step, the y-axis was aligned with the last 5 s of the baseline step, and the last 5 s of the association step was considered steady-state. Savitzky−Golay filtering was performed on the corrected curve to remove high-frequency noise from the data. Specific binding to M^pro^ was subtracted from the observed blank probes and reference control curves by selecting the “Double References” mode. A 1:1 binding model was assumed in the binding kinetics analysis.

### 2.5. Molecular Docking

Molecular docking of **G** compounds was performed using iGemdock software and following the instructions provided by the authors. The structures of **G1**−**G4** were drawn with ChemDraw 12.0 software and converted into Mol files with ChemBio3D Ultra software. The M^pro^ X-ray structure was retrieved from the Protein Data Bank (https://www.rcsb.org/structure/6W63 accessed on 7 July 2021) in the PDB format. For the docking experiments, amino acid residues including His41, Cys44, Leu141, Asn142, Gly143, Ser144, Cys145, His163, His164, Met165, Glu166, Arg188, and Gln189 were used as active sites. Following ligand and binding site preparation, virtual screening, post-screening analysis, and pharmacological interactions analysis, the docking conformation of the ligand was determined by selecting the pose with the lowest binding free energy. Structural analysis of system and graphics were performed using PyMOL2 2.3.3 (Accelrys Software Inc., San Diego, CA, USA) and Discovery Studio.

## 3. Results and Discussion

### 3.1. Synthesis of Fragment-Based **G**-Compounds

**Figure 2:**
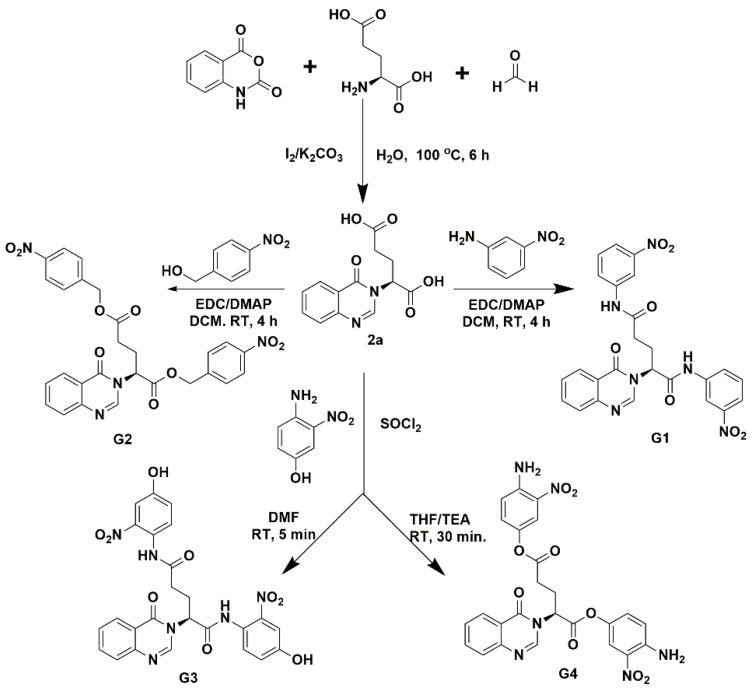
Synthesis of fragment-based compounds containing glutamine- or glutamic acid-derived peptidomimetic backbone, quinazolinone moiety, and nitro-functionalized phenyl derivatives.

Two types of fragment-based compounds were synthesized, with one glutamine-derived and one glutamate-derived peptidomimetic backbone (Figure 2). Additionally, either the side chain amide group of glutamine or the side chain carboxyl group of glutamic acid of the peptidomimetic backbones was attached to a nitro-functionalized phenyl derivative, because nitro groups of synthetic compounds often show binding interactions in predicted docking poses. The synthesized compounds (**G1**–**G4**) were separated by column chromatography and structurally characterized by high-resolution electrospray ionization mass spectrometry (HPLC/HR-ESI-MS) and ^1^H/^13^C nuclear magnetic resonance (NMR). These compounds were tested for their inhibitory effects on M^pro^ enzymatic activity.

### 3.2. M^pro^ Inhibition Assay

**Figure 3:**
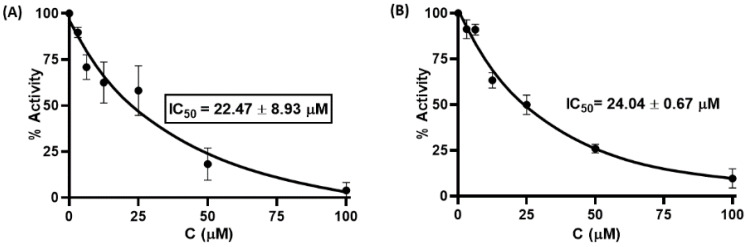
Inhibitory activity of serial dilutions of **G1** (**A**) and **G4** (**B**) against SARS-CoV-2 M^pro^.

The effect of the synthesized compounds on M^pro^ inhibitory activity was examined in an in vitro Förster resonance energy transfer (FRET) assay using Dabcyl-KNSTLQSGLRKE-Edans as the fluorogenic substrate peptide. This 12-amino acid fluorescent quenching paired peptide carries Dabcyl at the *N*-terminus and Edans at the *C*-terminus, which were paired, and when uncleaved, most of the energy emitted by Edans was quenched by Dabcyl. Recognition and cleavage of the peptide fragment by M^pro^ resulted in the separation of the two compounds and the release of fluorescence by Edans, which can be excited at 355 nm and detected at 460 nm. In parallel, **GC376**, a preclinical cysteine protease inhibitor that binds to the M^pro^ of the feline coronavirus (FCoV) was used as a positive control to validate the assay [21]. The **G1**–**G4** compounds were initially screened at 50 μM in an assay buffer (20 mM Tris, 100 mM NaCl, 1% DTT, 1% EDTA, and 1% DMSO) at a M^pro^ concentration of 50 nM. The results showed that two compounds (**G1** and **G4**) exhibited more than 50% inhibitory activity against M^pro^. We next quantified the inhibitory activities of different inhibitor concentrations (100, 50, 25, 12.5, 6.25, and 3.125 μM). As shown in Figure 3, **G1** and **G4** inhibited M^pro^ enzymatic activity in a dose-dependent manner with calculated IC_50_ values of 22.47 ± 8.93 μM and 24.04 ± 0.67 μM. These results suggest that glutamine- or glutamate-derived peptidomimetic backboned quinazolinone derivatives, combined with nitro-functionalized phenyl derivatives on the side chain or main chain, may play an important role in inhibiting M^pro^ enzymatic activity.

**Figure 4:**
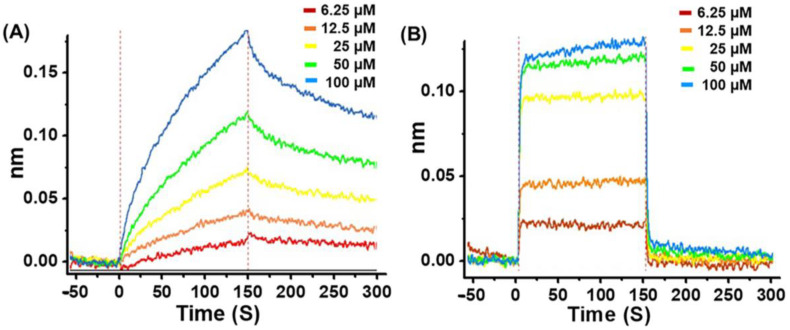
Bio-layer interferometry analysis of **G1** and **G4** with M^pro^. (**A**) Association and dissociation data of **G1** with M^pro^. (**B**) Association and dissociation data of **G4** with M^pro^.

To determine the inhibitory interaction of **G1** or **G4** with M^pro^, the kinetics of **G1**/M^pro^ and **G4**/M^pro^ association and dissociation were measured using the bio-layer interferometry (BLI) technique, an optical biosensing technique for real-time analysis biomolecular interactions without fluorescent labels [26,27]. Figure 4 shows the real-time binding and dissociation BLI kinetics of **G1**/M^pro^ and **G4**/M^pro^. Both displayed a similar equilibrium dissociation constant (*K*_D_ = *k*_off_/*k*_on_), at 2.60 × 10^−5^ M and 2.55 × 10^−5^ M, respectively, but exhibited distinct association/dissociation curves (Figure 4 and Table 1). **G1** bound logarithmically to the M^pro^-loaded probe in the binding segment of the kinetic cycle and slowly dissociated from the probe during the dissociation segment (Figure 4A). In contrast, **G4** bound strongly to the M^pro^-loaded probe in the first 5 s of the association segment, rapidly reached steady-state and dissociated from the probe in the first 5 s of the dissociation segment (Figure 4B). The *k*_on_ and *k*_off_ values for **G1** and **G4**, respectively, also demonstrate the same trend. These results suggest that **G1** may be a long-term potent inhibitor to M^pro^, while **G4** is a fast-acting inhibitor to the protease.

### 3.3. Pharmacological and Bioavailability of the Synthesized Compounds

The **G1**–**G4** were evaluated for physicochemical properties and bioavailability for potential oral active drugs using the Adsorption, Distribution, Metabolism and Excretion (ADME) assay from SwissADME (Table 2) [28]. Although **G1**–**G4** do not satisfy Lipinski’s rule of five due to their molecular weights exceeding 500 Da, the computational physicochemical properties can be compensated by intramolecular H-bonding and formulation that allow the significant expansion of the molecular weight (MW), the polar surface area (PSA), and the hydrogen bond acceptor atom (HBA) [20,29,30,31]. For example, there are many macrolides and peptide structures such as erythronolides, leucomycins, and vancomycins of antibacterial, antiviral, antifungal, immunosuppressive, and anticancer agents with an MW of >500 Da and high PSAs and HBAs that do not satisfy Lipinski’s rule of five but still observe excellent human bioavailability [29,30,31]. In addition, the hepatitis C virus NS3/4A protease inhibitor designed with the weak heptapeptide leader also has a linear or macrocyclic peptidomimetic structure with a molecular weight of >700 Da [32]. One explanation is that passive permeability across cell membranes is inversely proportional to the hydrodynamic radius of the compound; that is, it depends on the size and the shape of the compound. Thus, although the pharmacological data for the above compounds are beyond the rule of five, with poor solubility, cell permeability, metabolism, and toxicity, this can be compensated by intramolecular H-bonding and formulation that allow the significant expansion of the MW, the PSA, and the HBA [20,29,31 and references therein].

### 3.4. Molecular Docking

**Figure 5:**
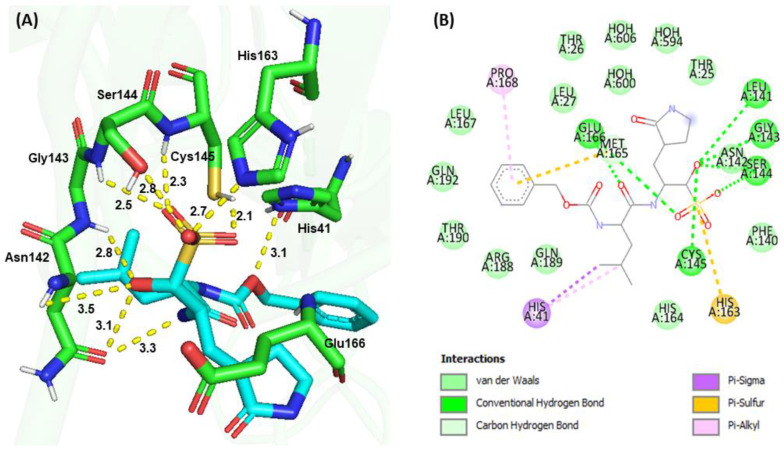
Molecular docking of **GC376** and SARS-CoV-2 M^pro^. (**A**) 3D interactions between **GC376** (Cyan) and M^pro^ (Green). (**B**) 2D interactions, including H-bonding, hydrophobic, π−sulfur, π−π, and π−alkyl interactions between **GC376** and M^pro^, identified by Discovery Studio.

Molecular docking is a popular drug discovery method often used to predict protein-ligand affinity and the stability of interactions before conducting experiments. To elucidate the structure−function relationship of M^pro^ inhibition with synthesized compounds, docking simulations of **GC376**, **G1**, and **G4** were performed and compared with the crystal structure of the **GC376**−M^pro^ complex [20,21]. Similar binding pocket and active site cavity packing, electrostatic, hydrogen bonding, and hydrophobic interactions were observed between the molecular docking and the X-ray structure of the **GC376**−M^pro^ complex, supporting the accuracy of the docking model (Figure 5). A binding affinity analysis indicated that **GC376** bound to the active site cavity of M^pro^ with a binding energy of −158.75 kJ/mol. The ligand **GC376** bound to the active site pocket Gly143-Ser144-Cys145 oxyanion hole and forms conserved H-bond interactions with the backbone amide donors of His163 and Glu166. Additionally, Cys145 interacted with His41 to form a catalytic dyad and formed hydrogen bonds and hydrophobic interactions with amino acid residues Thr25, His41, Phe140, Leu141, Asn142, Gly143, Ser144, Cys145, His163, His164, Met165, Glu166, Leu167, Pro168, Arg188, Gln189, Thr190, and Gln192 in the active site cavity. The glutamine surrogate ring at the P1 position of **GC376** was embedded into the S1 pocket and formed an H-bond with the Nε of the imidazole ring of His163 and the side chain carboxyl group of Glu166. The Leu at the P2 position was inserted into the S2 hydrophobic pocket consisting of Arg40, His41, Met49, Tyr54, Met165, Arg188, and Gln189, as reported by Fu et al. [20]. The carbonyl group on the benzyl ring at the P3 position of **GC376** was also inserted into the S1 binding site to form an H-bond with the backbone amide group of Glu166.

**Figure 6:**
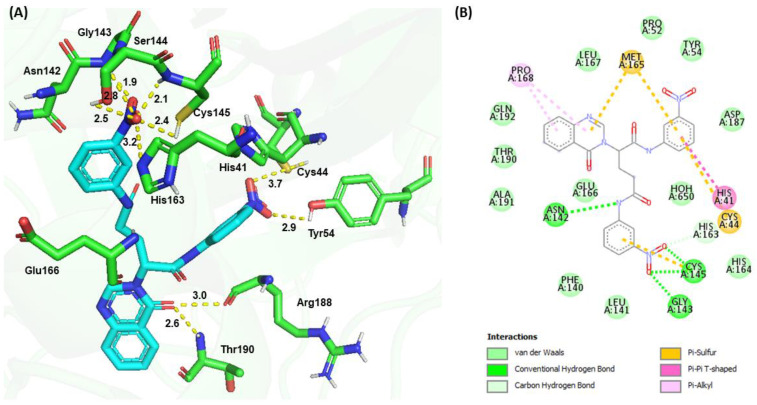
Molecular docking of **G1** and SARS-CoV-2 M^pro^. (**A**) 3D interaction between **G1** (cyan) and M^pro^ (green). (**B**) 2D interactions between **G1** and M^pro^, including H-bonding, hydrophobic, π−sulfur, π−π, and π−alkyl interactions, as determined by Discovery Studio.

The molecular docking of **G1** showed a binding energy of −187.80 kJ/mol. The −NO_2_ (1) group at the P1 position of **G1** formed H-bonds with the side chain sulfhydryl group (3.7 Å) of Cys44 and the phenolic group (2.9 Å) of Tyr54, while the −NO_2_ (2) group at the P2 position formed several H-bond interactions, including the main chain amide groups of Gly143 (1.9 Å), Ser144 (2.8 Å), and Cys145 (2.1 Å), the side chain sulfhydryl group (2.4 Å) of Cys145, and the hydroxyl group of Ser144 (2.5 Å), as well as the Nε of the imidazole ring (3.2 Å) of His163. The Nγ of the quinazolinone ring formed an H-bond with the backbone carbonyl group of Glu166 (Figure 6). The carbonyl group of the quinazolinone ring interacted with the backbone carbonyl group (3.0 Å) of Arg188 and formed an H-bond with the backbone amide group (2.6 Å) of Thr190. Additional π−sulfur interactions were observed between the quinazolinone ring and Met165, the aromatic ring at the P1 position and Cys44, and the aromatic ring at the P2 position and Cys145. The phenyl group at P1 interacted with the imidazole ring of His41 through a π−π interaction. Pro168 formed π−alkyl interactions with the quinazolinone ring.

**Figure 7:**
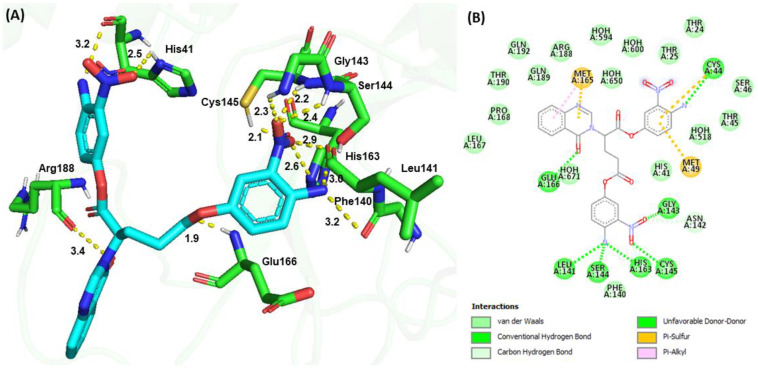
Molecular docking of **G4** and SARS-CoV-2 M^pro^. (**A**) 3D H-bond interactions between **G4** (cyan) and M^pro^ (green). (**B**) 2D interactions between **G4** and M^pro^, including H-bond, hydrophobic, π−sulfur, and π−π interactions, as determined by Discovery Studio.

Molecular docking revealed a binding energy of −188.67 kJ/mol for **G4** and a similar binding configuration to **G1**. The −NO_2_ (1) group and −NH_2_ (1) at the P1 position formed H-bonds with the Nδ of the imidazole ring (2.5 Å) and the carbonyl group (3.2 Å) of His41, respectively. The −NO_2_ (2) group at the P2 position formed H-bonds with the backbone amide groups of Gly143 (2.3 Å), backbone amide (2.4 Å), and carbonyl (2.9 Å) groups of Ser144; the backbone amide group (2.2 Å) and the side chain sulfhydryl group (2.1 Å) of Cys145; and the Nε (2.6 Å) of the imidazole ring of His163. The −NH_2_ (2) group at the P2 position formed an H-bond with the backbone carbonyl group (3.0 Å) of Leu141 and the backbone carbonyl group (3.2 Å) of Phe140. The carboxylate group of the peptidomimetic backbone formed an H-bond with the backbone amide group (1.9 Å) of Glu166 (Figure 7). The carbonyl group of the quinazolinone ring interacted with the backbone carbonyl group (3.4 Å) of Arg188. Additional π−sulfur interactions were observed between the quinazolinone ring and Met165 and between the aromatic ring at the P1 position and Cys44 and Met49. The phenyl moiety of the quinazolinone ring exhibited π−alkyl interactions with Met165. **G4** also exhibited numerous van der Waals interactions with Thr24, Thr25, His41, Thr45, Ser46, Met49, Phe140, Leu167, Pro168, Arg188, Gln189, Thr190, and Gln192.

Numerous in silico M^pro^ inhibitors have been identified; however, only a few in vitro experiments have been performed to validate virtual screening results [33,34,35]. Meanwhile, two clinical approved drugs, GSK-256066 and bicalutamide, showed more than 30% M^pro^ inhibitory activity at a concentration of 50 μM [36]. Coelho et al. reported a biochemical high-throughput screening of a 2,400-drug library and identified 13 inhibitors with IC_50_ values between 0.2 and 23 μM [37]. **GC376** is a pre-clinical inhibitor against feline infectious peritonitis coronavirus (FIPV) that inhibits M^pro^ in vitro with an IC_50_ of 0.15 ± 0.03 μM [20,21]. However, the side effects of **GC376**, such as delayed adult teeth development in animals, prevent it from being approved by the FDA as a M^pro^ inhibitor [38]. In this study, a series of glutamine- or glutamate-derived peptidomimetic backbone with quinazolinone moieties and nitro-functionalized derivatives were synthesized, and their inhibitory effects on the M^pro^ protease of SARS-CoV-2 were investigated. Among the synthesized **G**-compounds, **G1** and **G4** were determined to have good IC_50_ inhibitory activity in an approximately 20 μM range, indicating a comparable performance to the published results, although their oral bioavailability predictions exceeded that of Lipinski’s rule of five.

Previous studies have shown that the putative M^pro^ inhibitor **GC376** binds to the active site pocket Gly143-Ser144-Cys145 oxyanion hole and forms conserved H-bond interactions with the backbone amide donors of His163 and Glu166 [20,21]. Molecular docking of **G1** and **G4** with M^pro^ showed that both compounds showed similar binding configurations, namely the P1 moiety pointed to His41 and Cys44, the P2 moiety pointed to the Gly143-Ser144-Cys145 oxyanion hole, and formed electrostatic and hydrogen bonding interactions with amino acid residues around the cavity. Although −NO_2_ groups are used relatively conservatively in drug development due to their metabolic instability, nitro-containing ligands have been increasingly used as biological probes with appropriately positioned cysteine and acidic side chains to form covalent inhibitors with acceptable potencies and no apparent toxicity [23,25]. Interestingly, the −NO_2_ group at the P1 position pointed to Cys44, while the −NO_2_ group at the P2 position pointed to Cys145, which is reminiscent of the hypothesis of the formation of a targeted covalent inhibitor from an appropriately positioned weak electrophilic group and the active site nucleophilic residues of proteins. Perhaps the simultaneous introduction of −NO_2_ functional groups at the P1 and P2 positions could form stable bidentate chelating interactions with active site residues, thereby facilitating ligand binding and synergistically increasing inhibitory activity. Consistent with the observations was a dramatic drop of inhibitory activity when the nitrophenyl group at the P2 position was deleted. The quinazolinone moiety at the P3 position formed a π−sulfur or π−alkyl interaction with Met165, Glu166, Arg188, or Thr190 and enhanced its binding affinity.

## 4. Conclusions

Using a fragment-based strategy including a potential antiviral quinazolinone moiety, a glutamine- or glutamate-derived peptidomimetic backbone, and positioned nitro-functionalized phenyl groups to form targeted covalent inhibitors, we successfully synthesized and identified **G1** and **G4** to have in vitro M^pro^ inhibitory activity with IC_50_ values of 22.47 ± 8.93 μM and 24.04 ± 0.67 μM, respectively. **G1**/M^pro^ and **G4**/M^pro^ displayed similar *K*_Ds_ values of 2.60 × 10^−5^ M and 2.55 × 10^−5^ M, respectively, but exhibited different association/dissociation curves. The molecular docking results of **G1** and **G4** suggest that they bind to the same active region as **GC376** and may form targeted covalent ligand-protein complexes. These results support the new scaffold as a candidate for M^pro^ inhibition and advance anti-coronavirus drug research. Experimental validation to further confirm the inhibitory potential of these compounds against COVID-19 is ongoing.

## Figures and Tables

**Table 1:** Bio-layer interferometry analysis data of **G1**/M^pro^ and **G4**/M^pro^. *K*_D_ = *k*_off_/*k*_on_; *k*_on_—association constant; *k*_off_—dissociation constant; R^2^—coefficient of determination.

	*K*_D_ (M)	*k*_on_ (1/Ms)	*k*_off_ (1/s)	R^2^
**G1**	2.60 × 10^−5^ ± 1.49 × 10^−7^	1.25 × 10^2^ ± 4.99 × 10^−1^	3.25 × 10^−3^ ± 1.33 × 10^−5^	0.9913
**G4**	2.55 × 10^−5^ ± 7.59 × 10^−7^	1.62 × 10^4^ ± 3.50 × 10^2^	4.13 × 10^−1^ ± 8.44 × 10^−3^	0.971

**Table 2:** Physico-chemical properties of the synthesized compounds in terms of the drug likeness and the bioavailability score.

Name	Lipinski’s Rules					
	MW <500	HBA <10	HBD ≤5	Mlog *P* ≤4.15	Lipinski’s Volations	Bioavailability Score
**G1**	516.46	8	2	1.63	2	0.17
**G2**	546.48	10	0	2.31	2	0.17
**G3**	548.46	10	4	−0.13	2	0.17
**G4**	548.46	10	2	0.41	2	0.17

## Data Availability

The data presented in this study are available on request from the corresponding author.
